# Community structure in the phonological network

**DOI:** 10.3389/fpsyg.2013.00553

**Published:** 2013-08-27

**Authors:** Cynthia S. Q. Siew

**Affiliations:** Spoken Language Laboratory, Department of Psychology, University of KansasLawrence, KS, USA

**Keywords:** network science, mental lexicon, community structure, lexical processing, language acquisition, language evolution, phonology

## Abstract

Community structure, which refers to the presence of densely connected groups within a larger network, is a common feature of several real-world networks from a variety of domains such as the human brain, social networks of hunter-gatherers and business organizations, and the World Wide Web (Porter et al., 2009). Using a community detection technique known as the Louvain optimization method, 17 communities were extracted from the giant component of the phonological network described in Vitevitch (2008). Additional analyses comparing the lexical and phonological characteristics of words in these communities against words in randomly generated communities revealed several novel discoveries. Larger communities tend to consist of short, frequent words of high degree and low age of acquisition ratings, and smaller communities tend to consist of longer, less frequent words of low degree and high age of acquisition ratings. Real communities also contained fewer different phonological segments compared to random communities, although the number of occurrences of phonological segments found in real communities was much higher than that of the same phonological segments in random communities. Interestingly, the observation that relatively few biphones occur very frequently and a large number of biphones occur rarely within communities mirrors the pattern of the overall frequency of words in a language (Zipf, 1935). The present findings have important implications for understanding the dynamics of activation spread among words in the phonological network that are relevant to lexical processing, as well as understanding the mechanisms that underlie language acquisition and the evolution of language.

## Introduction

In the past decade or so, the application of graph-theoretic methods to model a variety of complex, large-scale real-world phenomena has burgeoned. Graph-theoretic approaches refer to the techniques developed by mathematicians to characterize and describe the topology or structure of a network (Watts and Strogatz, 1998; Watts, 2004). Researchers from a multitude of disciplines have applied these techniques to investigate large-scale networks such as the Internet (Yook et al., 2002), scientific collaborations (Barabási et al., 2002), the human brain (Bullmore and Sporns, 2009) and the mental lexicon (Steyvers and Tenenbaum, 2005; Vitevitch, 2008).

The tools of network science have been applied to study language by creating semantic networks constructed from either word association data or co-occurrence statistics, and networks of phonological word-forms. Applying graph theoretic methods to analyze language networks is a fast growing and particularly productive area of research. Previous work with respect to semantic networks have important implications for the cognitive mechanisms underlying language processing because they suggest that these mechanisms exploit network structure to facilitate the processing of language and retrieval of semantic knowledge. For instance, researchers have shown that the network structure of word associations is a superior predictor of human responses on a fluency task, which indicate that the search for a relevant response to a given cue is dependent on the link structure of the semantic network, as well as the relative importance of these links within memory (Griffiths et al., 2007). The network structure of word associations was also a good predictor of semantic similarity between pairs of seemingly unrelated words, which might indicate a common ontological organization of words and concepts across people (De Deyne et al., 2012). Work by Hills et al. (2009, 2010) on early semantic networks of children has also contributed considerable insight into the longitudinal development of the mental lexicon, especially in terms of the roles of different network growth mechanisms in language acquisition. Their results also showed that the network of words within a child's language learning environment is an important predictor of the words that children learn first, which have important implications for language acquisition.

The tools of network science have also been used to model the organization of phonological word-forms in the mental lexicon (Vitevitch, 2008). In a phonological network, nodes represent phonological word forms, and links connect words that are phonologically similar to each other. Two words are said to be phonologically similar or “phonological neighbors” of each other if the first word can be transformed into the second word by the substitution, addition or deletion of one phoneme (Luce and Pisoni, 1998). The phonological network examined in Vitevitch (2008) displayed the properties of a small-world network; that is, short average path length and high average clustering coefficient, features of network topology that have been commonly observed in other real world networks (Watts and Strogatz, 1998).

With regards to the phonological network, the clustering coefficient of a word, a network science measure that describes the local structure of a node, has been shown to influence lexical processing of spoken words (Chan and Vitevitch, 2009, 2010), as well as long and short-term memory processes (Vitevitch et al., 2012). These results are theoretically important because they place additional constraints on current models of spoken word recognition, which are unable to accommodate these findings as they do not explicitly take into account the role of network structure on lexical processing.

Taken together, prior work applying the theory and methods of network science to the study of language has been invaluable, as these studies have provided evidence for the psychological reality of the network structure of semantic and phonological networks, and showed that the nature of network structure has measurable influences on lexical processing and language acquisition.

The recent movement toward using network science to describe the overall structure of the lexicon contrasts with the traditional approach of psycholinguistic research which has typically focused on the lexical characteristics of individual words, such as word frequency and neighborhood density. Previous work from the network science approach has shown that language networks and other complex networks share several *macro-level* features, such as being “small-world” (i.e., short average path lengths and large average clustering coefficients; Steyvers and Tenenbaum, 2005; Vitevitch, 2008), and possessing a degree distribution which approximates a power law (Steyvers and Tenenbaum, 2005; Hills et al., 2009; but not for the phonological network, where the degree distribution is better fit by a truncated power law, see Arbesman et al., 2010b). In contrast, previous psycholinguistic research largely concentrated on investigating the influence of *micro-level* lexical variables (i.e., characteristics of individual words) on spoken word recognition and production (e.g., Savin, 1963; Broadbent, 1967; Taft and Hambly, 1986; Luce and Pisoni, 1998; Vitevitch and Luce, 1998, 1999; Garlock et al., 2001).

There exist theoretically important reasons to investigate the *meso-level* of the phonological network. This paper represents a first step in this direction by extracting and analyzing the community structure of the phonological network of words. In the following paragraphs I provide examples of how community detection techniques have been used to study other complex networks, and briefly show how these have enhanced our understanding of the structure and dynamics of networks. Then, to motivate the present work, predictions with respect to the community structure of phonological word forms and potential theoretical significance of applying community detection to the phonological network will be briefly discussed.

Community structure refers to the presence of several smaller groups of nodes contained in a larger network. These smaller groups form such that there are many connections among nodes within a group, but few connections between nodes in different groups (Newman and Girvan, 2004; Newman, 2006). This phenomenon has caught the attention of network scientists because it has been generally observed that communities are a ubiquitous feature of real-world networks in a variety of domains, such as the structure of a human brain (Wu et al., 2011), social networks of hunter-gatherers, business organizations and Facebook friends (Porter et al., 2009), and the World Wide Web (Newman, 2004). The observation that real-world networks tend to divide naturally into smaller networks has led to the general hypothesis that this natural division reflects the presence of a hierarchical structure, where larger communities consist of smaller communities in an iterative pattern (Ravasz and Barabási, 2003), or the encapsulation of functions or local interactions in a complex system (Girvan and Newman, 2002; Newman and Girvan, 2004). For instance, community structure may indicate the presence of protein clusters with similar biological functions in a protein-interaction network (Ravasz et al., 2002) or reflect the underlying social organization and hierarchy of societies (Porter et al., 2009).

The vast majority of the literature has focused on using the tools of network science to delineate the network topology of a complex system. Ultimately, however, the goal is to understand how network structure influences the dynamics and functioning of a network. Community detection analyses have the potential to reveal details of network structure that may not be observable at the coarse, top-most level of analysis, nor by examining the individual nodes that comprise the system (Lancichinetti et al., 2010; Onnela et al., 2012). In a study investigating the spread of disease in a network with community structure reflecting the social make-up of a society, Kitchovitch and Liò (2011) showed that disease tends to spread more efficiently within the community than across communities, revealing a more detailed understanding of the spread of a disease that would not be possible if only the dynamics of the entire network or of individuals in the system were analyzed. Similarly, uncovering community structure in the phonological network can enhance our understanding of how dynamic processes underlying word recognition and production—specifically, the spreading of activation among word nodes—are affected by the community structure of the network.

In the phonological network, undirected and unweighted links are placed between words that are phonologically similar to each other. Therefore, phonologically similar words that share common phonological segments tend to cluster together and are likely to form a community within the network. The presence of communities in the phonological network could reflect the grouping of these phonological segments in English. As evidenced from high clustering coefficients, there exist naturally occurring clusters within the phonological network in Vitevitch (2008) because the distributions of phonemes that make up any single word are not random. Community detection can reveal the presence of these clusters which may enhance our understanding of the underlying phonological structure of language.

Phonemes are widely recognized as the smallest units of a language [Sapir, 1933; but see Savin and Bever (1970) for evidence against the perceptual reality of phonemes], and words are formed by stringing together sequences of phonemes. A long-standing research topic in psycholinguistics deals with the phonological structure of words in a language, and early researchers have studied how the distribution of phonological segments among various word types influences lexical processing of these words (Greenberg and Jenkins, 1964; Landauer and Streeter, 1973). More recently, it has been shown that certain phonological characteristics of words, such as phonotactic probability (Vitevitch and Luce, 1998, 1999) influences the speed and accuracy of lexical retrieval. Phonological segments (in the simplest case, a pair of phonemes) that occur more frequently than other phonological segments are said to be of high phonotactic probability. Nonwords containing phonological segments of high phonotactic probability are recognized more quickly than nonwords containing phonological segments of low phonotactic probability (Vitevitch and Luce, 1998, 1999; Luce and Large, 2001).

As mentioned earlier, real-world networks tend to naturally divide into smaller sub-graphs and display a hierarchical structure that can be observed at the mesoscopic level. Phonological word forms in the mental lexicon can be said to display such a hierarchical structure as well. For example, the presence of short words (such as “cat”) embedded in longer words (such as “catalog” or “concatenate”), word clusters that share common onsets (e.g., “cat,” “catalog” and “caterpillar”) and words that share common rimes (e.g., “cat,” “bat,” and “rat”) have been well-documented and investigated by psycholinguists (e.g., Marslen-Wilson, 1987; McQueen, 1996; Norris et al., 2002; McQueen and Sereno, 2005). In particular, Marslen-Wilson's cohort theory (1987) posits that recognition of a word occurs when the phonological sequence of that word begins to diverge from the phonological sequence of other words sharing the same initial phonological sequence. In this theory, cohorts consist of words that share a common onset and become smaller as more phonological information becomes available over time (Marslen-Wilson, 1987); this is analogous to a hierarchy where a large group of words can be subdivided into smaller groups depending on the degree of phonological overlap (from the initial phoneme) between words. On the other hand, there is also evidence showing that word recognition is facilitated when participants are primed with words that share the same rime, and it has been argued that the phonological saliency of a group of words with the same rime prompted a biased processing strategy among participants (Norris et al., 2002; McQueen and Sereno, 2005). Therefore, community detection methods can reveal community structure that reflects grouping of phonological word forms by cohort or by rime (or even potentially reflect both kinds of grouping) which may afford deeper insights into how the overall phonological structure of words influences and facilitates lexical retrieval processes.

Examining the community structure of the phonological network may also have important implications for various aspects of psycholinguistics and language sciences. Here I briefly speculate on how community structure in the phonological network may enhance our understanding of lexical processes. Despite the fact that the average adult mental lexicon consists of 30,000–80,000 words (Aitchison, 2012), people are able to recognize and produce words rapidly and efficiently. This ability to retrieve word forms efficiently from a relatively dense or highly clustered large network of words strongly suggests that lexical retrieval mechanisms may exploit the community structure of the phonological network to facilitate rapid and accurate word recognition and production.

Community detection could also reveal how network structure at differing levels of the network influences lexical processing in distinctive ways. The finding that high probability segments facilitates lexical processing is seemingly at odds with the neighborhood density effect observed for spoken words, where words with several phonological neighbors are in fact *less* accurately and more slowly recognized compared to words with fewer neighbors (Luce and Pisoni, 1998; Goh et al., 2009). Words belonging to dense neighborhoods by definition also contain high probability segments. Phonological similarity appears to be simultaneously implicated in the *facilitatory* effects of probabilistic phonotactics and *inhibitory* effects of neighborhood density in spoken word recognition.

Investigating the structure of the phonological network at various levels of the network could help us understand the opposing effects of phonotactics and density on spoken word recognition. Neighborhood density reflects a micro-level measure of network structure, as it is simply the degree of a node. On the other hand, community structure measures network structure at the meso-level because it assesses the connectivity of words beyond that of a word's local neighborhood. It is possible that phonotactic effects on processing emerge as a consequence of the community structure of the phonological network. Therefore, phonotactic and neighborhood effects may not be entirely contradictory if one considers the connectivity of phonological word forms at various levels of a network. This approach is somewhat analogous to the adaptive resonance framework proposed by Vitevitch and Luce (1999), in order to account for their finding that facilitatory effects of phonotactic probability were observed when processing nonwords and competitive effects of neighborhood density were observed when processing words. This framework consisted of sublexical and lexical types of representations which have dissociable and distinct effects on lexical processing, and arise depending on the nature of the processing task.

Previously, researchers such as Landauer and Streeter (1973) and others (Frauenfelder et al., 1993; Schiller et al., 1996; Kessler and Treiman, 1997) studied the distributional properties of phonological segments in language using straightforward metrics (such as frequency counts of individual phonemes and biphones). These previous approaches, however, were limited by the computational power and technology available at the time. Now, the tools of network science and community detection techniques can be used to answer intriguing questions about the underlying phonological structure of a language. Current metrics of phonotactic probability have focused on segment and biphone co-occurrence probabilities (e.g., Vitevitch and Luce, 2004). However, Auer and Luce (2005) noted that there is a need to develop metrics of phonotactic probability to detect larger phonological sequences (i.e., that are longer than a pair of phonemes) and assess their influence on speech perception and production. Investigating the phonological segments of words that belong to specific communities could allow us to extract longer phonological sequences that frequently co-occur among words and ultimately determine if listeners are sensitive to these larger segments in speech processing and whether the processing of words which contain these segments is facilitated.

To recapitulate, the aim of the present paper is to uncover community structure of the phonological network described in Vitevitch (2008). To this end, a common community detection technique known as the Louvain optimization method was applied to the giant component of the phonological network. For comparison of the mean lexical characteristics and biphone distributions of the observed communities, the same words from the giant component were randomly assigned to communities (of sizes comparable to the observed communities) to provide a “baseline” for the measures of interest. The lexical characteristics analyzed in this paper include word length, subjective familiarity, word frequency, neighborhood density, neighborhood frequency, positional and biphone probability, and age of acquisition. These characteristics were chosen because they are known to influence the speed and accuracy of lexical processing in a variety of psycholinguistic experimental paradigms such as lexical decision, perceptual identification and word shadowing (Savin, 1963; Broadbent, 1967; Taft and Hambly, 1986; Luce and Pisoni, 1998; Turner et al., 1998; Vitevitch and Luce, 1998, 1999; Garlock et al., 2001; Ghyselinck et al., 2004; Goh et al., 2009). Past work investigating the statistical properties of words has also focused on comparing word length, frequency and neighborhood density (e.g., Zipf, 1935; Frauenfelder et al., 1993). In order to relate the present analyses back to previous work, the same variables are also investigated here. As words belonging to the same community may also share similar phonological properties, mean positional and biphone probabilities are also analyzed because these variables represent commonly used measures of the phonemic properties of words, and have been shown to influence lexical processing as well (Vitevitch and Luce, 1998; Vitevitch et al., 1999).

With respect to the phonological properties of words in communities, it follows that words belonging to the same community should share similar phonological characteristics as the phonological network was constructed based on phonological similarity. However, it is not entirely obvious if words belonging to the same community will also share similar lexical characteristics. Furthermore, the phonological and lexical properties of words in each community may depend on the size of the community. If one conceptualizes the network as containing a self-similar structure where communities consist of smaller communities (which consist of even smaller communities), the largest community may resemble the “giant component” of the phonological network whereas smaller communities resemble lexical islands. The phonological network in Vitevitch (2008) consisted of a giant component of 6,508 words, several lexical islands (small networks of words that are not connected to the giant component) and hermits (individual words that are not connected to any other words). The giant component consisted of words which were shorter in length, of higher frequency and higher neighborhood density (i.e., degree) than words in the lexical islands. Analogously, one might predict that larger communities in general consist of shorter words of higher frequency and higher density than words from smaller communities.

## Material and methods

### The phonological network

The phonological network in Vitevitch (2008) was constructed from approximately 20,000 words obtained from the Hoosier Mental Lexicon (Nusbaum et al., 1984). In this network, each node corresponded to a word's phonological transcription obtained from the Merriam-Webster Pocket Dictionary. An undirected and unweighted link (or edge) was added between two nodes if the two words were phonologically similar.

Phonological similarity was defined as the substitution, addition or deletion of one phoneme at any position between two given words (Greenberg and Jenkins, 1964; Landauer and Streeter, 1973; Luce and Pisoni, 1998). This measure is commonly used in the literature to calculate the phonological neighborhoods of a given word, and has a long history in psycholinguistics (Landauer and Streeter, 1973; Luce and Pisoni, 1998). Furthermore, this metric has been shown to be a psychologically valid method in assessing phonological similarity—when asked to produce a word that sounds similar to a given word, participants tend to produce words that differ from the given word by one phoneme (Luce and Large, 2001). The word /kæt/ (“cat”) would have a phonological neighborhood consisting of /bæt/ (“bat”), /skæt/ (“scat”) and /æt/ (“at”), among other words. In the phonological network, these words are also known as “phonological neighbors” of the word /kæt/ and would be connected via undirected and unweighted links to the node representing /kæt/.

Vitevitch (2008) found that the network consisted of a giant component of 6,508 words, lexical islands (words that are connected to each other, but not to any other words in the large component), and lexical hermits (words that had no phonological neighbors, known as isolates in the network science literature). In the present analyses, I extracted the community structure of the large component of 6,508 words. Islands and hermits were excluded from the analyses because by definition, each island and hermit constitutes a “community” of its own, so community detection conducted on these words is unlikely to yield meaningful or interpretable results.

### Community detection

Modularity, *Q*, measures the density of links inside communities as compared to links between communities^1^ (Newman, 2006; Fortunato, 2010), and is mathematically defined as

$$Q=\frac{1}{2m}\sum_{i,\;j}\left[A_{ij}-\frac{k_{i}k_{j}}{2m}\right]\delta (c_{i},c_{j})$$

where *A*_*ij*_ represents the adjacency matrix of the weights of the edge between nodes *i* and *j, k*_*i*_ is equal to the sum of the weights of the edges attached to node *i, c*_*i*_ is the community to which node *i* is assigned, *c*_*j*_ is the community to which node *j* is assigned, the δ function δ(*c*_*i*_, *c*_*j*_) is 1 if *c*_*i*_ = *c*_*j*_ and 0 otherwise, and $m=\frac{1}{2}\sum_{ij}A_{ij}$. Since the present network has unweighted edges, *A*_*ij*_ is simply reduced to a matrix with constants, and *k*_*i*_ and *k*_*j*_ is equal to the number of edges attached to node *i* and node *j* respectively.

Modularity is also used as a measure of the quality of partitions resulting from community detection methods (Fortunato, 2010). Positive *Q* values that are close to the maximum value of 1.0 indicate the presence of high quality communities,^2^ where the density of links within communities is high relative to the density of links between communities. *Q* values of large real-world networks such as the Internet and cellular phone networks, as well as smaller social networks such as Zachary's karate club, range from 0.42 to 0.78 (Blondel et al., 2008). Although some variability exists among various kinds of complex networks, the fact that all of these networks have positive *Q* values imply that the community structure of these networks is very robust, which implies that the partitions delineating communities in the network are highly distinct.

The Louvain method is a modularity optimization or “greedy” optimization approach. The algorithm consists of two phases that are repeated iteratively. In the first phase each node is assigned to one community such that there are as many communities as there are nodes. The gain in modularity is evaluated by removing node *i* from its community and placing it in the communities of its neighbors *j*, and node *i* is placed in the community which yields the greatest gain in modularity. This is done for the rest of the nodes in the network. In the second phase, a new network is built where nodes are the communities found in the first phase. Both phases are iterated until the highest possible value of *Q* is obtained. Although the output of this algorithm varies depending on the order in which the nodes are considered, Blondel et al. (2008) indicates that this does not have a significant influence on the quality of the partitions produced by the algorithm.

There exists a host of other community detection methods such as centrality or edge betweenness based techniques and dynamic methods such as clique percolation (e.g., Derényi et al., 2005). However, what distinguishes the Louvain method from others lies in its simple yet intuitive algorithm, which is reminiscent of the self-similar nature of complex networks (Blondel et al., 2008). The algorithm also integrates the idea of hierarchy within a network, as communities of communities are built at each pass (Blondel et al., 2008). Related to this is the notion of resolution limit in modularity optimization approaches. The resolution limit refers to the observation that whether small communities can be successfully extracted using modularity optimization methods is dependent on the size of the network and the extent of interconnectedness of its communities (Fortunato and Barthelemy, 2007; Porter et al., 2009). To address this issue, the resolution parameter can be specified in order to extract communities at a particular level of the network's community structure, such that not too many or too few communities are extracted from the network. For the phonological network, the Louvain algorithm was conducted 5 times at various resolutions, from 1.0 to 5.0 in 1.0 increments. A *t*-test comparing the modularity values at resolutions 1.0 (average *Q* = 0.675) and 2.0 (average *Q* = 0.667) showed that these values did not statistically differ from each other, and were significantly higher than those obtained with 3.0 or higher resolutions. A table summarizing *Q* values and number of communities yielded by the algorithm at each resolution is included in the Supplementary Materials. This indicated that the quality of communities extracted using resolutions of 1.0 and 2.0 were not only similar but also very high. In this case, a resolution of 2.0 was used because it yielded a smaller number of communities at a slightly higher level of hierarchy (Lambiotte et al., 2008), in order to facilitate statistical analyses and the interpretation of results. Note that using the communities extracted using a resolution of 1.0 did not result in qualitative differences in the interpretation of the following analyses.

Although there exists a wide variety of community detection methods to determine the presence of community structure in networks [see Porter et al. (2009) or Fortunato (2010) for a review of these algorithms], the Louvain method was chosen because of the high quality of communities detected using this method, as well as short computational times (Blondel et al., 2008). Although the choice of the Louvain community detection method is admittedly somewhat arbitrary, several researchers have noted that most community detection methods yield very similar results despite having different algorithms, differing on very nuanced details such as whether communities are allowed to overlap or not (Porter et al., 2009). Therefore, it is unlikely that the communities obtained using the Louvain method are the result of an artifact from using a particular community detection algorithm. The Louvain algorithm is readily available in Gephi (Bastian et al., 2009).

### Random communities

A baseline model of “random” communities was constructed in order to provide a point of comparison for the lexical characteristics of the communities extracted from the giant component by the detection algorithm. The random communities were generated by randomly assigning words from the giant component to the *same number* of communities with the *same sizes* as those extracted using the Louvain method. This permits a meaningful and unbiased comparison of the “real” communities that were generated using the community detection algorithm and “random” communities that were obtained via arbitrarily grouping words into communities of the same sizes. This randomization procedure is commonly used to generate baseline communities in studies that have investigated community structure in other complex networks (e.g., Traud et al., 2008).

## Results

### Communities in the phonological network

Using a resolution of 2.0 the community detection algorithm found 17 communities, with a mean size of 382.82 (*SD* = 249.29) nodes per community. As shown in Table 1 below, the sizes of the 17 communities varied, ranging from 31 to 697 words. Communities were relabeled such that community 1 represented the smallest community and community 17 represented the largest community. Modularity, *Q*, was 0.655, a moderately large positive value, which implies the presence of robust community structure in the phonological network. Notably, this value lies between the range of modularity values obtained for various networks as computed in Blondel et al. (2008).

**Table 1:** **Community sizes for 17 communities extracted from the phonological network**.

**Community**	***N***
1	31
2	37
3	38
4	85
5	127
6	271
7	278
8	348
9	397
10	520
11	543
12	544
13	625
14	626
15	654
16	687
17	697
MEAN	382.82
SD	249.29

In order to assess if the observed community structure is indeed a genuine feature of the phonological network, two Erdõs-Renyi (ER) graphs with 6,508 nodes were generated in Pajek (Batagelj and Mrvar, 1998). ER Graph A was generated with the same mean degree as that of the real network (29627 edges/6508 nodes = 4.55). This produced a random graph with a mean degree of 4.535, but with only 14,757 edges, much fewer than the 29,627 edges in the real network. Therefore, a second graph, ER Graph B, was generated with a higher mean degree of 9.105, which produced a random graph with a mean degree of 9.198 and 29,929 edges. Note that these two ER graphs were generated because of the constraints involved in generating a random ER graph that had the same number of edges and same mean degree as that of the phonological network. This constraint is due to the fact that the degree distribution of language networks is skewed, whereas the degree distribution of an ER graph resembles a Poisson distribution (Erdõs and Renyi, 1961; Newman, 2003). Therefore, two different graphs were generated. The Louvain community detection algorithm (using the same parameters used to detect communities in the phonological network) was applied to both ER graphs. ER Graph A yielded 78 communities with a modularity of 0.232 and ER Graph B yielded 2 communities with a modularity of 0.0.

Most notably, the modularity values of the randomly generated networks (*Q* = 0.232 and 0.0) were much smaller than that of the phonological network (*Q* = 0.655). This indicates that the communities extracted from these random graphs are of lower quality than the communities extracted from the phonological network. Recall that a large modularity value (close to 1) implies that the community structure not only exists within the network but also that the structure is highly robust (Blondel et al., 2008). The high modularity value of the phonological network relative to the random networks strongly suggests that the language network consists of tightly connected communities and therefore this community structure is worth investigating further. In the following section, statistical analyses will be conducted on the mean lexical characteristics and phonotactic properties of these communities.

### Lexical characteristics in the communities

As a basis of comparison in the analyses of various lexical characteristics, the community membership of words in the giant component was randomized to form “random communities” (which should not be confused with the Erdõs-Renyi “random networks” used in the previous section Communities in the Phonological Network). To distinguish the communities found in the giant component using the Louvain method, I will use the phrase “real communities.”

To investigate how the 17 real (and random) communities might be distinguished from each other, 1-way between-group ANOVAs (with the 17 communities as the independent variable) were conducted to compare the mean lexical characteristics of words in the real and random communities. These lexical characteristics (i.e., the dependent variable in the ANOVAs) include word length, subjective familiarity, word frequency, neighborhood density, neighborhood frequency, positional and biphone probability, and age of acquisition. Table 2 summarizes the mean of all lexical variables for each of the 17 real and random communities. Note that the communities have been relabeled such that community 1 represents the smallest community and community 17 represents the largest community.

**Table 2:** **Summary of descriptive statistics for lexical characteristics of each real and random community**.

	**Word length**		**Familiarity**		**Word frequency**		**Neighborhood density**		**Neighborhood frequency**		**Positional probability**		**Biphone probability**		**Age of acquisition**	
	**Mean**	**SD**	**Mean**	**SD**	**Mean**	**SD**	**Mean**	**SD**	**Mean**	**SD**	**Mean**	**SD**	**Mean**	**SD**	**Mean**	**SD**
**REAL COMMUNITIES**																
1	4.452	0.961	5.634	1.475	1.576	0.617	2.258	1.094	1.687	0.433	0.04406	0.01522	0.00471	0.00236	10.533	2.818
2	4.946	0.780	5.289	1.808	1.489	0.756	2.649	1.549	1.373	0.422	0.05677	0.00902	0.00648	0.00168	10.393	3.227
3	4.342	0.745	4.818	1.960	1.207	0.322	2.342	1.146	1.230	0.236	0.04527	0.01288	0.00354	0.00216	11.781	2.378
4	5.129	0.985	6.132	1.190	1.583	0.675	2.918	1.642	1.673	0.562	0.05092	0.01014	0.00930	0.00340	10.538	3.032
5	4.654	0.938	5.689	1.531	1.442	0.549	2.984	1.830	1.514	0.434	0.06086	0.00991	0.00638	0.00185	10.597	3.063
6	3.686	0.799	6.093	1.387	1.712	0.848	10.635	8.669	1.830	0.408	0.03885	0.01346	0.00273	0.00217	8.898	3.112
7	3.878	0.823	6.210	1.286	1.677	0.828	10.227	7.770	1.722	0.405	0.04359	0.01075	0.00309	0.00152	8.215	2.814
8	4.422	0.787	6.014	1.422	1.644	0.701	6.017	4.751	1.706	0.421	0.04662	0.00993	0.00405	0.00168	9.374	3.073
9	4.128	0.972	5.875	1.562	1.857	0.866	8.814	7.783	1.989	0.538	0.05455	0.01470	0.00545	0.00285	9.028	3.389
10	4.123	0.865	5.810	1.539	1.629	0.791	7.656	6.677	1.695	0.461	0.04627	0.01218	0.00426	0.00271	9.347	3.100
11	3.825	0.855	5.956	1.512	1.729	0.792	11.449	9.546	1.815	0.429	0.04384	0.01450	0.00288	0.00214	8.878	3.414
12	4.268	1.029	5.915	1.485	1.688	0.769	5.044	4.436	1.787	0.498	0.05089	0.01668	0.00553	0.00348	9.697	3.129
13	4.160	0.959	6.013	1.415	1.711	0.842	9.483	8.449	1.795	0.479	0.05206	0.01299	0.00526	0.00271	8.953	3.118
14	3.682	0.928	5.956	1.425	1.865	0.918	11.229	8.763	1.948	0.476	0.04164	0.01355	0.00268	0.00192	9.091	3.341
15	4.142	0.854	6.060	1.351	1.663	0.792	9.096	8.236	1.744	0.457	0.04861	0.01490	0.00449	0.00291	8.847	3.080
16	3.905	0.903	6.072	1.407	1.860	0.897	10.531	9.121	1.944	0.493	0.04282	0.01468	0.00290	0.00229	8.829	3.311
17	4.022	0.891	6.006	1.412	1.827	0.852	11.389	9.456	1.953	0.469	0.04730	0.01216	0.00396	0.00259	8.944	3.098
Overall	4.058	0.937	5.974	1.448	1.734	0.828	9.100	8.289	1.822	0.483	0.04704	0.01430	0.00411	0.00281	9.106	3.206
**RANDOM COMMUNITIES**																
1	4.161	0.820	5.489	1.749	1.782	0.967	7.032	5.666	1.774	0.432	0.04303	0.01659	0.00342	0.00287	10.576	3.374
2	3.946	0.575	5.773	1.659	1.697	0.905	9.703	8.553	1.705	0.375	0.04960	0.01334	0.00397	0.00207	8.370	2.661
3	4.079	0.997	5.794	1.590	1.737	0.769	9.184	8.577	1.925	0.604	0.05061	0.01329	0.00426	0.00304	8.813	3.615
4	4.012	0.893	6.163	1.342	1.733	0.900	8.129	7.773	1.873	0.567	0.04393	0.01509	0.00391	0.00270	8.724	3.056
5	4.213	0.879	5.875	1.499	1.695	0.804	6.906	6.560	1.855	0.500	0.04981	0.01342	0.00472	0.00281	9.427	3.091
6	3.993	1.004	5.965	1.454	1.711	0.824	9.745	8.767	1.838	0.468	0.04651	0.01421	0.00391	0.00251	8.877	3.006
7	4.047	0.984	5.914	1.452	1.738	0.849	8.727	8.101	1.848	0.483	0.04706	0.01462	0.00419	0.00290	9.129	3.329
8	4.126	0.940	5.885	1.486	1.674	0.812	8.580	8.053	1.797	0.488	0.04714	0.01464	0.00427	0.00283	9.031	3.198
9	4.058	0.904	5.956	1.469	1.727	0.846	9.164	8.443	1.812	0.508	0.04742	0.01402	0.00404	0.00274	8.948	3.211
10	4.071	0.960	6.007	1.416	1.751	0.872	9.269	8.397	1.814	0.482	0.04755	0.01483	0.00432	0.00288	9.166	3.221
11	4.064	0.965	5.978	1.460	1.860	0.907	9.637	8.710	1.832	0.492	0.04821	0.01441	0.00421	0.00293	8.917	3.278
12	4.053	0.926	5.924	1.465	1.692	0.774	9.158	8.286	1.778	0.442	0.04705	0.01414	0.00404	0.00277	9.142	3.122
13	4.080	0.913	5.998	1.477	1.719	0.793	9.421	8.421	1.853	0.478	0.04729	0.01399	0.00409	0.00282	9.071	3.102
14	4.061	0.927	5.979	1.451	1.720	0.810	8.941	8.137	1.801	0.477	0.04703	0.01445	0.00412	0.00278	9.178	3.178
15	4.034	0.964	6.045	1.386	1.761	0.824	8.821	8.088	1.850	0.486	0.04562	0.01451	0.00400	0.00284	9.219	3.249
16	4.051	0.920	5.993	1.450	1.728	0.824	9.403	8.362	1.810	0.474	0.04739	0.01424	0.00419	0.00303	9.129	3.304
17	4.030	0.938	5.998	1.413	1.713	0.799	9.019	8.287	1.826	0.494	0.04612	0.01372	0.00389	0.00255	9.159	3.238
Overall	4.058	0.937	5.974	1.448	1.734	0.828	9.100	8.289	1.822	0.483	0.04704	0.01430	0.00411	0.00281	9.106	3.206

A significant ANOVA indicates that the lexical characteristic that is being compared is significantly different across the 17 communities in either the real or the random communities. The results of the 1-way ANOVAs are summarized in Table 3. Note that as a number of Levene's tests of homogeneity of variances were significant (see Table 3), the assumption of homogeneity of variances is violated. Because ANOVA is not robust to heteroscedasticity of variances when group sizes are unequal (Maxwell and Delaney, 2004), alternative *F*-statistics using corrected degrees of freedom were calculated where relevant for the omnibus *F*-tests.

**Table 3:** **Summary of statistical analyses for real and random communities**.

**Lexical characteristics**	***F*-test**	**Linear contrast**
**REAL COMMUNITIES**		
Word length	*F* (16, 261) = 35.12, *p* < 0.001	*F* (1, 104) = 100.00, *p* < 0.001
Familiarity	*F* (16, 199) = 3.28, *p* < 0.001	*F* (1, 124) = 16.39, *p* < 0.001
Word frequency	*F* (16, 234) = 12.73, *p* < 0.001	*F* (1, 116) = 46.80, *p* < 0.001
Neighborhood density	*F* (16, 271) = 166.87, *p* < 0.001	*F* (1, 1666) = 1412.58, *p* < 0.001
Neighborhood frequency	*F* (16, 288) = 39.34, *p* < 0.001	*F* (1, 113) = 158.59, *p* < 0.001
Positional probability	*F* (16, 183) = 53.25, *p* < 0.001	*F* (1, 84.4) = 11.03, *p* < 0.01
Biphone probability	*F* (16, 259) = 101.72, *p* < 0.001	*F* (1, 121) = 116.48, *p* < 0.001
Age of acquisition	*F* (16, 214) = 9.73, *p* < 0.001	*F* (1, 91.3) = 49.58, *p* < 0.001
**RANDOM COMMUNITIES**		
Word length	*F* (16, 6490) = 0.59, *p* = 0.90	
Familiarity	*F* (16, 6490) = 0.731, *p* = 0.76	
Word frequency	*F* (16, 6490) = 1.15, *p* = 0.30	
Neighborhood density	*F* (16, 252) = 1.77, *p* < 0.05	*F* (1, 145) = 2.50, *p* = 0.12
Neighborhood frequency	*F* (16, 6490) = 1.20, *p* = 0.26	
Positional probability	*F* (16, 6490) = 1.859, *p* < 0.05	*F* (1, 6490) = 0.11, *p* = 0.74
Biphone probability	*F* (16, 6490) = 1.332, *p* = 0.17	
Age of acquisition	*F* (16, 5569) = 0.94, *p* = 0.52	

*(1) Corrected F-tests were conducted using corrected degrees of freedom if Levene's test of homogeneity of variances was significant. (2) The linear trend post-hoc contrast was conducted only if the omnibus F-test was statistically significant*.

**Figure 1:**
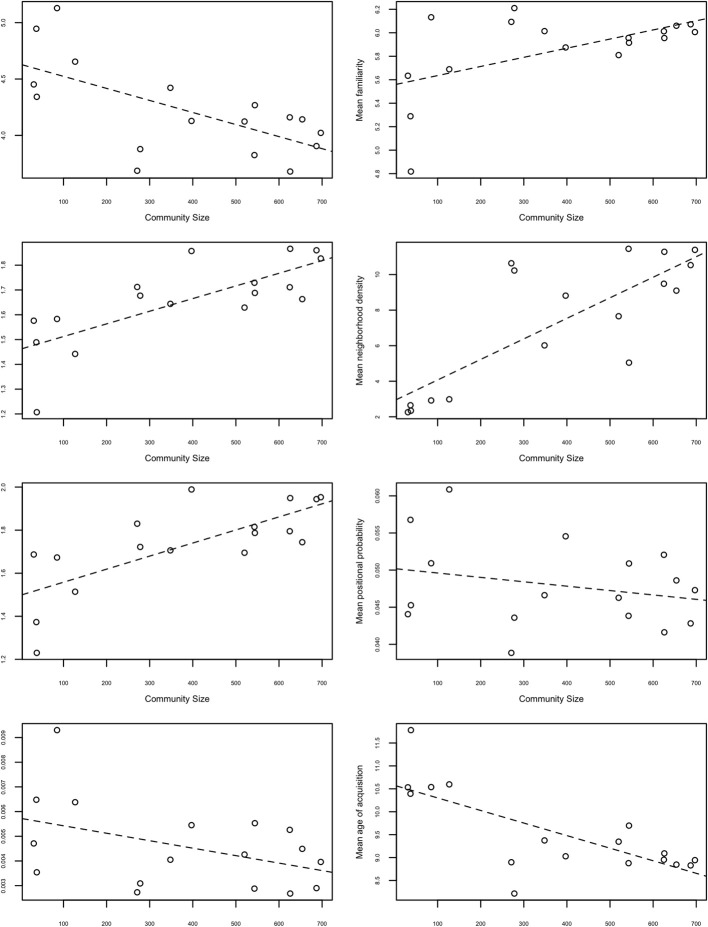
**Plots of mean lexical characteristics of each community against community sizes.** The x-axis represents the number of words residing in each community. The y-axis represents the mean lexical characteristics for each of the 17 communities. The dashed line represents the best-fit line.

If the *F*-omnibus test was significant, then *post-hoc* linear trend analyses and correlational analyses were conducted in order to gain additional insight into the relationships between community size and lexical characteristics. If the *post-hoc* linear trend analysis and correlation between the lexical characteristic and community size are significant, this implies that the magnitude of the mean lexical characteristics of each of the 17 communities varies depending on the size of the community. Figure 1 shows the relationship between these lexical characteristics and community size.

#### Word length

Word length was measured by counting the number of phonemes in a given word. The 1-way ANOVA was significant, *F* (16, 261) = 35.12, *p* < 0.001, indicating that some communities contained mostly long words and other communities contained mostly short words. The *post-hoc* linear contrast [*F* (1, 104) = 100.00, *p* < 0.001] and the correlation between mean length and community size were also significant (*r* = −0.653, *df* = 15, *p* < 0.01), indicating that larger communities tend to consist of shorter words (i.e., the words contain fewer phonemes), whereas smaller communities tend to consist of longer words.

#### Subjective familiarity

Subjective familiarity values were obtained on a 7-point scale, such that words with higher familiarity scores were perceived to be more familiar (Nusbaum et al., 1984). The 1-way ANOVA was significant, *F* (16, 199) = 3.28, *p* < 0.001, indicating that some communities contained mostly familiar words and other communities contained mostly unfamiliar words. The *post-hoc* linear contrast [*F* (1, 124) = 16.39, *p* < 0.001] and the correlation between mean familiarity and community size were also significant (*r* = 0.560, *df* = 15, *p* < 0.05), indicating that larger communities tended to consist of highly familiar words, whereas smaller communities tended to consist of less familiar words.

#### Word frequency

Word frequency refers to how often a given word occurs in a language, and log-base 10 of the raw frequency counts from Kučera and Francis (1967) were used in the present analyses. The 1-way ANOVA was significant, *F* (16, 234) = 12.73, *p* < 0.001, indicating that some communities contained mostly high frequency words and other communities contained mostly low frequency words. The *post-hoc* linear contrast [*F* (1, 116) = 46.80, *p* < 0.001] and the correlation between mean word frequency and community size were also significant (*r* = 0.753, *df* = 15, *p* < 0.001), indicating that larger communities tended to consist of more frequent words, whereas smaller communities tend to consist of less frequent words.

The finding that larger communities tend to consist of frequent and short words is reminiscent of Zipf's (1935) more general observation that short words tend to be also very frequent words in a language. Thus, the overall structure of the language appears to be reflected in the communities observed in the present study, much like the structure of a fractal is observed at both large and small scales.

Furthermore, given Zipf's (1935) additional observation that there are few high frequency words and many low frequency words in a language, it is interesting to note that most of these high frequency words are found in the largest communities. These large communities consisting of high frequency words may reflect sections of the giant component where a large amount of cognitive processing occurs compared to other parts of the lexical network.

#### Neighborhood density

Neighborhood density refers to the number of words that are phonologically similar to a given word (Luce and Pisoni, 1998). Phonological similarity is defined as the substitution, addition, or deletion of one phoneme in a given word to form a phonological neighbor. Note that this is identical to the criteria used to decide if two words in the network used in the present analysis should be connected by an edge or not, and is therefore equivalent to the network science term *degree*. The 1-way ANOVA was significant, *F* (16, 271) = 166.87, *p* < 0.001, indicating that some communities contained mostly words of high neighborhood density (or high degree) and other communities contained mostly words of low neighborhood density (or low degree).

The *post-hoc* linear contrast [*F* (1, 1666) = 1412.58, *p* < 0.001] and the correlation between mean neighborhood density and community size were also significant (*r* = 0.802, *df* = 15, *p* < 0.001), indicating that larger communities tended to consist of high-density words (or nodes with high degree), whereas smaller communities tend to consist of low-density words (or nodes with low degree). This result is in line with the idea that communities are simply sub-graphs of the original network (Ravasz and Barabási, 2003). The largest communities would be analogous to the giant component of a network, and smaller communities are analogous to islands. As the giant component is a very densely connected section of the network compared to the connectivity of disconnected components (islands), one might expect words in the larger communities to be of higher degree than words in the smaller communities.

#### Neighborhood frequency

Neighborhood frequency is the mean word frequency of a word's phonological neighbors. Log-base 10 values of word frequency counts were obtained from Kučera and Francis (1967). The 1-way ANOVA was significant, *F* (16, 288) = 39.34, *p* < 0.001, indicating that some communities contained mostly words with high frequency neighbors and other communities contained mostly words with low frequency neighbors. The *post-hoc* linear contrast was also significant [*F* (1, 113) = 158.59, *p* < 0.001] and the correlation between mean neighborhood frequency and community size were also significant (*r* = 0.741, *df* = 15, *p* < 0.001), indicating that larger communities tended to consist of words with high frequency neighbors, whereas smaller communities tended to consist of words with low frequency neighbors. Again, this is consistent with the previously mentioned finding that high frequency words tend to occur in large communities.

#### Phonotactic probability

The phonotactic probability of a word refers to the probability that a segment occurs in a certain position of a word (positional segment probability), and the probability that two adjacent segments co-occur (biphone probability; Vitevitch and Luce, 1998). These values were obtained from the Phonotactic Probability Calculator^3^ (Vitevitch and Luce, 2004). The 1-way ANOVA for positional probability was significant, *F* (16, 183) = 53.25, *p* < 0.001, indicating that some communities contained words of high positional probability and other communities contained words of low positional probability. The *post-hoc* linear contrast was significant, *F* (1, 84.4) = 11.03, *p* < 0.01; however, the correlation between mean positional probability and community size was not significant, *p* = 0.32.

The 1-way ANOVA for biphone probability was significant, *F* (16, 259) = 101.72, *p* < 0.001, indicating that some communities contained words of high biphone probability and other communities contained words of low biphone probability. The *post-hoc* linear contrast was significant, *F* (1, 121) = 116.48, *p* < 0.001; however, the correlation between mean biphone probability and community size was marginally significant, *p* = 0.08.

It is important to note that phonotactic probability on its own does not tell us the underlying phonological structure of each community, as phonotactic probability is a value that indicates the frequency of occurrence of a phoneme in a particular position (or the co-occurrence of two phonemes in the case of biphone probability) in a given language. It is possible for two communities to have similar mean phonotactic probabilities, but different types or combinations of phonemes and biphones could have contributed to this value. Therefore, to investigate the phonological structure of communities, additional analyses on the biphone frequencies for each community were conducted (see section “Raw Biphone Counts”).

#### Age of acquisition

Age of acquisition refers to the age at which a particular word was learned (e.g., Ghyselinck et al., 2004). Age of acquisition ratings are typically obtained by asking participants to indicate the age at which a particular word was learned (e.g., Cortese and Khanna, 2008; Kuperman et al., 2012). Ratings for 5,568 words were obtained from the Kuperman et al. (2012) megastudy. As ratings were not available for the other 940 words in the giant component, these words were not included in these analyses.

The 1-way ANOVA for age of acquisition ratings was significant, *F* (16, 214) = 9.73, *p* < 0.001, indicating that some communities contained words with high age of acquisition ratings and other communities contained words with low age of acquisition ratings. The *post-hoc* linear contrast [*F* (1, 91.3) = 49.53, *p* < 0.001) and the correlation between mean age of acquisition ratings and community sizes were also significant (*r* = −0.739, *df* = 15, *p* < 0.001), indicating that larger communities tended to consist of words with low age of acquisition ratings, whereas smaller communities tended to consist of words with high age of acquisition ratings. Given the well-documented observation that high frequency words tend to be words that are also acquired earlier in life (Ghyselinck et al., 2004; Kuperman et al., 2012), as well as the finding that high frequency words tend to reside in larger communities, it is perhaps not surprising that larger communities tend to consist of words with low age of acquisition ratings (i.e., acquired at a younger age). Nevertheless, this is a potentially important finding because it suggests that the larger communities are formed earlier than smaller communities, and could have implications for understanding language acquisition and growth dynamics of a language network (Steyvers and Tenenbaum, 2005).

### Random communities

In summary, ANOVAs and *post-hoc* linear trend analyses for all lexical characteristics were significant for real communities. Turning to the random communities, only the ANOVAs for neighborhood density and positional probability were significant, both *F*s < 1.86, both *p*s < 0.05, however, *post-hoc* linear trend analyses were not significant, both *F*s < 2.50, both *p*s > 0.12.

The absence of a significant linear trend for the random communities strongly suggests that the significant ANOVAs for neighborhood density and positional probability may be spurious. To assess this possibility, 4 additional sets of random communities were generated in the same manner and 1-way ANOVAs were conducted on them. None of the ANOVAs on the 4 new sets of randomly generated communities were significant^4^.

The fact that most of the ANOVAs for the random communities were not significant whereas all ANOVAs for the real communities were significant implies that the communities extracted by the community detection algorithm are not simply random groupings of words, but are capturing important relationships among words in the phonological network, such as the finding that larger communities tended to consist of shorter words of high frequency and neighborhood density as compared to smaller communities.

Note that the correlations between community sizes and community means of lexical characteristics reported for the real communities are consistent with the observed patterns reported in previous work (e.g., Zipf, 1935; Landauer and Streeter, 1973; Frauenfelder et al., 1993). For instance, larger communities tend to contain shorter words and more frequent words, which is the same pattern obtained by Zipf's (1935) analysis of the overall frequency of words in a language—words that frequently occur in corpora are also short words. This strongly suggests that these patterns may have implications for lexical processing and language evolution.

### Raw biphone counts

As mentioned in the Introduction, Auer and Luce (2005) pointed out that current measures of phonotactic probability might not allow us to detect and hence assess the influence of longer phonological segments on lexical processing. To investigate whether communities consist of words which contain similar phonological segments, raw counts of biphones found in words belonging to the same community, henceforth referred to as raw biphone counts, were obtained from each of the 17 real and random communities. Note that these raw biphone counts represent a measure of how often a particular biphone occurs *within each community*. On the other hand, the positional and biphone probabilities that were analyzed in the ANOVAs above represent how often a particular segment occurs at a certain word position and the overall probability of occurrence of those biphones within a corpus of words respectively (Vitevitch and Luce, 2004), and hence do not directly indicate whether similar phonological segments occur in the same community. It should be emphasized that the raw biphone counts obtained for each community are not position-specific and do not represent overall frequencies in a language, unlike commonly used phonotactic measures in the literature.

Two-way Kolmogorov-Smirnov (K-S) tests were conducted to compare the raw biphone counts found in the real and random communities of the same size. The results are summarized in Table 4. All K-S tests were significant, *D*s > 0.10, *p*s < 0.05, except for Communities 11 and 17, and the K-S test for Community 16 was marginally significant, *D* = 0.09, *p* = 0.077. These results indicated that the raw biphone counts of communities obtained using community detection methods were significantly different from the raw biphone counts of randomly generated communities.

**Table 4:** **Summary of Kolmogorov-Smirnov tests for raw biphone counts of real and random communities**.

**Community**	***D*-statistic**	***p*-value**
1	0.366	0.001^**^
2	0.288	0.001^**^
3	0.287	0.007^**^
4	0.296	<0.001^***^
5	0.309	<0.001^***^
6	0.175	0.002^**^
7	0.174	0.001^**^
8	0.136	0.005^**^
9	0.149	0.002^**^
10	0.107	0.025^*^
11	0.077	0.191
12	0.119	0.004^**^
13	0.127	0.004^**^
14	0.066	0.295
15	0.127	0.002^**^
16	0.086	0.077^+^
17	0.071	0.202

*** p < 0.001,

** p < 0.01,

* p < 0.05,

+ p < 0.10.

**Figure 2:**
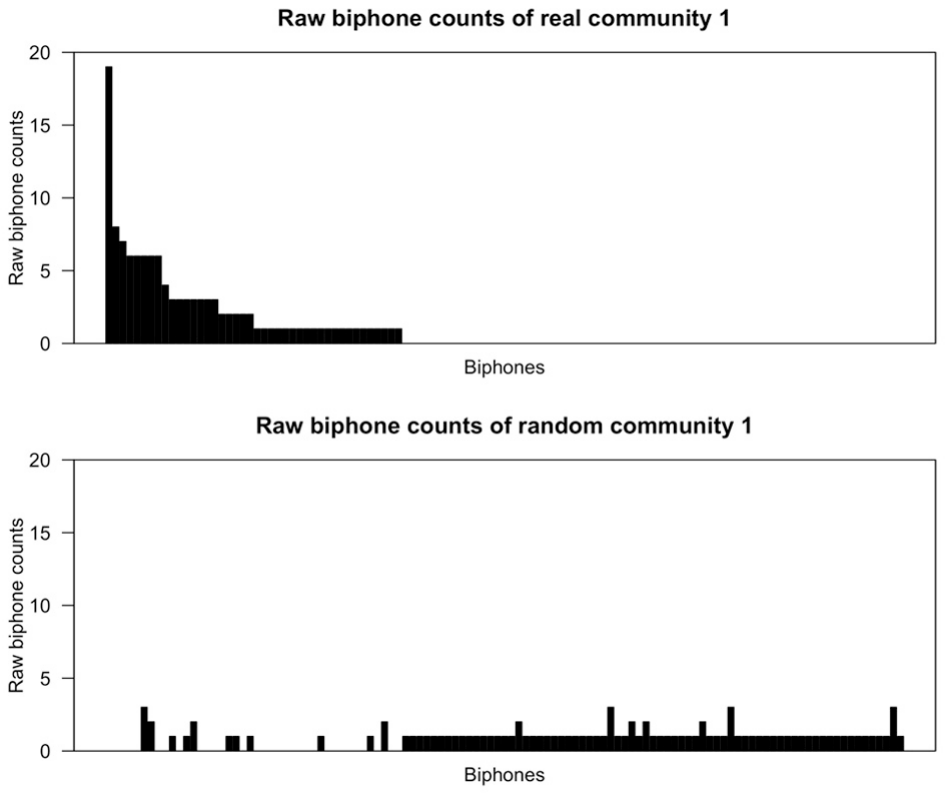
**Raw biphone counts of real and random community 1.** The x-axis represents the different biphones found within these communities and the biphones (on both x-axes) were arranged based on their frequency of occurrence in the *real* community in descending order.

**Figure 3:**
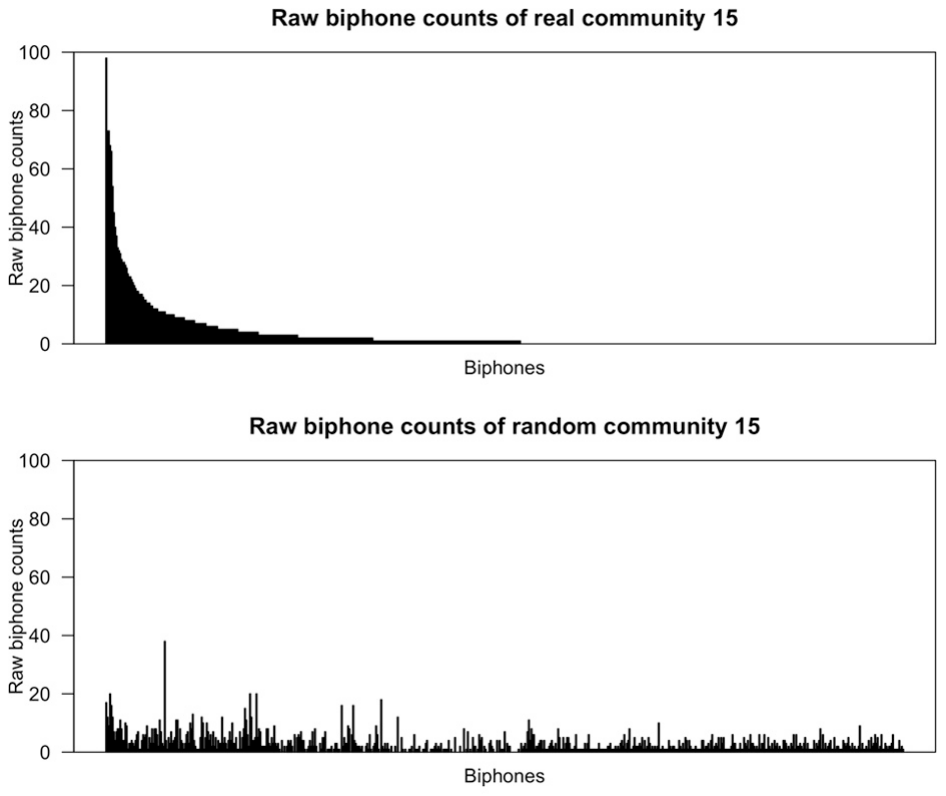
**Raw biphone counts of real and random community 15.** The x-axis represents the different biphones found within these communities and the biphones (on both x-axes) were arranged based on their frequency of occurrence in the *real* community in descending order.

Figures 2, 3 show the raw biphone counts from the real and random communities 1 and 15. In these figures the sequence of biphones on the x-axis is the same for both real and random communities, and arranged (in decreasing order) by their frequency in the real community. Two things are clear from the figures. One, random communities contain a large number of different biphones compared to the real communities. Second, the raw counts of biphones found in real communities are much larger than the same biphones in random communities. Taken together, this strongly suggests that communities in the phonological network consist of words with certain phonological segments.

From a visual inspection of Figures 2, 3, it is clear that certain biphones are overrepresented in the real communities compared to random communities. It is interesting to note that there are relatively few biphones that occur frequently within a community, and a large number of biphones that occur rarely. Furthermore, this pattern was observed in all 17 real communities, but not in randomly generated communities. This pattern is also reminiscent of Zipf's (1935) finding that within a language there are few words that occur at very high frequencies but many words that occur less frequently. Despite the fact that the biphone distributions of each community consist of different biphones, it appears that biphone distributions at the community level mirror the overall frequency-of-occurrence pattern of words in a language.

Strikingly, the most frequent biphone pairs in real communities can be concatenated to form longer phonological segments. For instance, in community 1, the most frequently occurring biphones are /ɨn/, /bɨ/ and /ᴈb/, which can be concatenated to form a longer phonological segment, /ᴈbɨn/ (“urban”) that is then found in other words in that community, such as *urban, turbine* and *bourbon*. Similarly, in community 15, the most frequently occurring biphones are /ŋk/, /Iŋ/ and /ɹI/, which can be concatenated to form a longer phonological segment, /ɹIŋk/ (“rink”) that is then found in other words in that community, such as *brink, drink* and *wrinkle*. Thus, a large proportion of words in these communities contain these particular phonological segments and words in a community may simply be phonological variants of each other to varying degrees.

## Discussion

Using the Louvain method in Gephi, 17 communities were extracted from the giant component of the phonological network in Vitevitch (2008). Modularity, *Q*, was 0.655, which is much higher than the modularity values of the random ER networks and indicates the presence of strong community structure in the phonological network.

Additional analyses were conducted for both real and random communities to compare various lexical characteristics of words in different communities. Generally, ANOVAs and *post-hoc* linear trend analyses were significant for real communities but not for random communities. This indicated the presence of mean differences in lexical characteristics of words belonging to different communities, and linear contrasts suggested that the pattern of these differences was related to the size of the community. The results of these analyses were consistent with the prediction that larger communities tended to consist of short, frequent words of high degree, whereas smaller communities tended to consist of longer, less frequent words of low degree. Although ANOVAs were conducted separately for each lexical variable, these variables are by no means independent of each other; in fact, the present findings are consistent with previously observed patterns of correlations between various lexical variables (e.g., Zipf, 1935; Frauenfelder et al., 1993).

Raw biphone counts of real and random communities were also obtained and compared to uncover underlying patterns of phonological segments present in the extracted communities. K-S tests comparing real and random communities were significant for the majority of the 17 communities, indicating that the number of different phonological segments, as well as their raw counts (which represent the number of occurrences of that segment within a community), found in real communities were significantly different from that of random communities. The pattern that there are relatively few biphones that occur very frequently within communities and a large number of biphones that occur rarely is reminiscent of the pattern of the overall frequency of words in a language (Zipf, 1935). In addition, it should be highlighted that communities do not appear to be organized exclusively by cohorts or rimes, linguistic constructs that are commonly studied in the psycholinguistic literature (e.g., Marslen-Wilson, 1987; Norris et al., 2002). It is interesting to note that these linguistic constructs are not explicit features that are “build into” the organization of the phonological network.

Psycholinguistic research has traditionally focused on studying how various lexical characteristics of individual words, such as word frequency and neighborhood density, influence various aspects of lexical processing (e.g., Luce and Pisoni, 1998). Indeed, the existing literature has shown that these micro-level characteristics exert measurable and robust effects on spoken word recognition and production (e.g., Savin, 1963; Luce and Pisoni, 1998; Vitevitch and Luce, 1999). On the other hand, recent applications of network science to the study of both semantic and phonological language networks have revealed that the macro-level characteristics, such as average path length, average clustering coefficient and degree distribution, of these networks resemble that of other complex networks (e.g., Steyvers and Tenenbaum, 2005; Vitevitch, 2008).

Although both of these approaches have revealed important aspects about the micro- and macro-level of the phonological network, the present approach of applying a community detection method to this network has exposed the presence of robust community structure in the phonological network. Community structure can be viewed as the meso-level of the network, which describes the connectivity of the network at an intermediate level, rather than at the level of individual nodes or at the level of the entire network. As most complex networks comprise of a hierarchy of larger and smaller communities (Ravasz and Barabási, 2003), there exist different layers and levels of connectivity within the network that includes the more frequently studied micro- and macro-levels. Below I provide some examples of how the present findings with respect to the meso-level of the phonological network can provide deeper insights into lexical processing and language acquisition, beyond that of traditional psycholinguistic variables, as well as implications for the evolution of natural language.

### Implications for lexical processing

Community structure of a network can inform the dynamics of the spread of information within various networks (Lancichinetti et al., 2010; Kitchovitch and Liò, 2011; Wu et al., 2011). Similarly, the presence of community structure in the phonological network may be useful in explaining the dynamics of the spread of activation among words and how these dynamics influence lexical retrieval.

According to the spreading activation mechanism described in Chan and Vitevitch (2009; see also Vitevitch et al., 2011), when a word is activated, activation spreads to phonological neighbors of that word, and activation can also spread from these phonological neighbors back to the word that was initially activated. Such a mechanism has been used to explain why words with high clustering coefficients are more slowly recognized than words with low clustering coefficients. As activation becomes trapped within a densely connected local structure, it is difficult for the word with high clustering coefficient to “stand out” among other phonologically similar words and be subsequently recognized (Chan and Vitevitch, 2009).

With respect to community structure, it is possible that activation tends to be trapped within a community via the same mechanism described above, especially as words within communities are, by definition, more densely connected to each other than to words of other communities. There are some potential implications for lexical access.

If one conceptualizes lexical retrieval as a search problem within long-term memory, analogous to searching for a “patch” (a cluster of items) in memory to retrieve a target item from (Hills et al., 2012), then higher activation levels of words within one community compared to other communities in the network can facilitate lexical retrieval by narrowing the search space of the entire network to a smaller community. In fact, this could be a possible explanation of the observed phonotactic effects in speech perception and production—since communities tend to consist of words that share similar phonological segments, recognition of a target word that shares these same segments could be facilitated because of the higher overall activation levels of the community that the target word belongs to. On the other hand, words that contain segments of low phonotactic probability may not be recognized as quickly because other words that share those less common segments do not constitute a robust community within the network.

Conceiving phonotactic effects as an emergent property of the network's community structure could resolve the contradiction observed between facilitatory phonotactic effects and inhibitory neighborhood density effects in spoken word recognition. As mentioned earlier, these effects are contradictory because words that belong to dense neighborhoods also tend to contain common phonological segments. It is possible, however, that these effects arise at different levels or resolutions of the network—community structure at the meso-level reflects the grouping of words depending on their phonological segments, whereas neighborhood density reflects the degree of a word, or the number of phonological neighbors, which is a micro-level feature that captures the nature of a word's local network structure. This distinction between different resolutions of the network is akin to the framework of sublexical and lexical types of representation proposed by Vitevitch and Luce (1999), who suggested that different experimental tasks might emphasize the processing of either sublexical or lexical representations. An alternative but analogous way of understanding the discrete effects of phonotactic probability and neighborhood density could involve specifying the “resolution” of the lexical processes elicited by the experimental task, which could indicate the level of the phonological network that is emphasized during processing. The present finding that phonotactic probability “emerged” from the meso-level organization of *lexical* forms in the phonological network strongly suggests that phonotactic probability and density are *not* distinct, disparate features of phonological word forms; rather, the effects of phonotactic probability and neighborhood density arise depending on which level, or resolution, of the network that is emphasized by the processing task.

Another particularly striking finding that is worth noting is the finding that larger communities tend to consist of shorter words of high frequency and high density. The fact that high frequency words tend to reside in large communities implies that a large proportion of activation is primarily occurring at a particular region of the network because these words are frequently activated and retrieved. Since these high frequency words also tend to be of high neighborhood density (i.e., degree), this further implies that a substantial proportion of activation is trapped within this region as several other phonological neighbors also compete for recognition. It is interesting to note that these high frequency words are grouped together in large communities despite there being relatively few high frequency words as compared to low frequency words within the lexicon, in accordance with Zipf's (1935) law. Based on the spreading activation mechanism proposed by Chan and Vitevitch (2009), such an organization does not appear to be very efficient for lexical processing as it is difficult for any single word to “stand out” from other words that belong to the same community. On the other hand, words which belong to larger communities are also acquired relatively early in life. It is possible that acquiring certain words earlier affords them a processing advantage, for example, by making their meaning easily accessible (Brysbaert et al., 2000), so that these words can be easily retrieved in spite of their network structure. These are interesting and important hypotheses that deserve to be studied in greater detail.

Finally, the presence of larger phonological sequences that consist of more than just two phonemes lends some credence to the hypothesis put forward by Auer and Luce (2005) who speculated that such sequences could influence speech perception and production. Stimuli could be selected from the communities observed in the present analyses to empirically test the hypothesis put forward by Auer and Luce (2005).

### Implications for language acquisition

Although the present work has implications for other aspects of lexical processing such as understanding speech errors and learning of new words or a second language, it is beyond the scope of this paper to discuss the implications for all of these areas. Here I discuss a second area of lexical processing in which the present findings could have important implications for—language acquisition.

Community structure could potentially contribute toward our understanding of how language networks grow and change over time. Hills et al. (2009, 2010) have shown that the structure of the learning environment plays an important role in language acquisition. In addition, Hills et al. (2010) report that phonological neighbors play a role in predicting the order of acquisition of nouns. Furthermore, recent work by Beckage et al. (2011) found that the tendency for late talkers to acquire semantically novel words relative to known words could have resulted in the less “small world-like” structure of late talkers' early semantic networks compared to their typically developing peers.

Based on the above research findings, it is plausible that the early stages of language acquisition are crucial in the development of robust communities which ultimately promotes the growth of a cohesive language network that allows for rapid and efficient lexical processing to occur. The present finding that larger communities tend to consist of words that are learned earlier in life further suggests that larger communities may be among the first to develop in language acquisition, and may be a precursor for developing a robust language network. In addition, the finding that words containing similar phonological segments tend to belong to the same communities could lead to theoretically motivated predictions about the phonological properties of words that children tend to learn first, and how these might be different for children with language disorders or learning impairments. For instance, words that share similar phonological segments may tend to be acquired at about the same time in order to form the foundation of a new community within the phonological network. This is not inconceivable given that songs, limericks and nursery rhymes are important features of a child's early language learning environment. This could possibly further motivate the design of language learning programs or protocols that dictate which words should be acquired first (based on their phonological properties and community membership), which could help children with language or learning disorders establish robust community structure and subsequently a robust language network. It should be noted that these are speculations on my part, and additional research is required to address these interesting hypotheses.

### Implications for language evolution

As mentioned in the Introduction, communities are of special interest to network scientists because they are said to be signatures of naturally evolved real networks (Clune et al., 2013; Ravasz and Barabási, 2003). In particular, it has been shown computationally that modularity and communities arise naturally in a network when evolutionary processes take into account the *cost* of creating new connections (Clune et al., 2013). With respect to language evolution I suggest that there are two types of costs involved in the creation of new words; the first refers to phonotactic constraints and the second refers to communicative constraints of a speaker and a listener.

All languages are known to exhibit a property known as combinatorial phonology, where meaningless units (phonemes) can be combined to form meaningful units (morphemes) (Hockett, 1960). This property is important for language evolution because it results in the creation of all words of a language from simple combinations of a small number of phonemes (Hockett, 1960; Tria et al., 2012). This small number of phonemes relative to the large and possibly infinite number of words that could exist in a given language implies that phonemes were combined with other phonemes to form longer segments known as morphemes so that an unlimited number of referents (represented by different words) could be communicated between people without incurring excessive cognitive and memory cost (Nowak et al., 1999; Zuidema and De Boer, 2009). However, the combinatorial nature of phonology does not imply that all phoneme combinations are possible.

In English, not all phoneme combinations are legal, representing a phonotactic constraint on the creation of new words because of all possible words that could be formed by combining different phonemes, only a subset of those (which obey phonotactic rules) would constitute viable candidates for a “new” word. This is consistent with the finding that nonwords containing phonological segments of high phonotactic probability tend to be rated as very “wordlike,” i.e., these nonwords are highly possible words in a language (Frisch et al., 2000; Bailey and Hahn, 2001). In the present study, groups of similar phonological segments were observed within communities that were extracted from the phonological network. These segments might constitute the morphemes that are the result of the combination of different phonemes, which could represent the first important step in language evolution—combinatorial phonology. The presence of community structure in the phonological network, where words belonging to the same community share similar phonological segments and are essentially variants of each other, supports the findings of previous research on the emergence of morphology from phonology (Hockett, 1960; Tria et al., 2012).

The way in which language has evolved also needs to take into account the communicative constraints that arise when people communicate with each other. An “ideal” language would be one that consists of words which are very phonologically distinct from all other known words as this would minimize communication errors—although it may reduce production efficiency because a speaker has to be able to articulate a wide variety of different phonological sequences. However, real languages tend to contain several words that are phonologically very similar to known words (e.g., Landauer and Streeter, 1973; Frauenfelder et al., 1993) and, at least in English, these phonologically similar words are structurally organized within the community structure of the phonological network. From the perspective of a listener, the existence of several phonologically similar words may result in more errors of lexical retrieval or at least reduce processing efficiency (Frauenfelder et al., 1993), as these words are connected to many other words and compete for recognition within the phonological network. Nevertheless, it is possible that community structure within the language network provides some form of scaffolding for lexical processing, thereby increasing efficiency of lexical retrieval. This may be especially important if each of these phonologically similar words maps onto a different semantic referent, as in English. In comparison, greater morphological similarity exists among Spanish words compared to English words (Arbesman et al., 2010a), which could explain the finding that phonologically similar Spanish words are recognized more quickly than less phonologically similar Spanish words (Vitevitch and Rodríguez, 2005), compared to the inhibitory neighborhood density effect in English words.

Therefore, the presence of community structure in the phonological network may constitute preliminary evidence for the hypothesis that a language network evolves in a way that takes into account the competing needs of the listener and speaker, that also strikes a balance between polysemy (differentiation of meanings) and phonological similarity of words in a given language (Ferrer i Cancho and Solé, 2003).

## Conclusions

In the present paper, community detection methods revealed the presence of communities in the phonological network, and also uncovered novel aspects of the phonological network, such as (1) the finding that larger communities consist of short, frequent words of high degree and low age of acquisition ratings whereas smaller communities consist of longer, less frequent words of low degree and high age of acquisition ratings, (2) the similarity of the pattern of biphone distributions within communities to the pattern of frequency-of-occurrence of words in a language, and (3) the clustering of similar phonological segments in each community. These novel findings were observed using a community detection method that considers the structure of a network at an intermediate level, rather than at a purely global or local level. Therefore, even though similar relationships between the lexical characteristics of words have been found in previous studies (e.g., Zipf, 1935; Landauer and Streeter, 1973; Frauenfelder et al., 1993), the present findings are still significant because they relate to the *mesoscopic level* of the network, whereas the patterns found in prior work were of the *overall* relationship of words in the lexicon.

These findings also have important implications with respect to understanding the dynamics of the spread of activation within the phonological network, language acquisition, as well as the nature of language evolution. In particular, the presence of community structure within the phonological network could be said to be a “signature” of language evolution, which could further indicate the different ways in which language could have evolved, in order to take into account articulation costs to maximize communicative efficiency, or to allow for the emergence of morphology from phonology. Although these conclusions are admittedly somewhat speculative in nature, the present findings are significant because they not only show that community structure exists within the phonological network, but also more importantly that this community structure reflects the grouping of phonological word forms with similar lexical characteristics and contain similar phonological segments at a mesoscopic level. Future research can be directed toward investigating these intriguing speculations in greater detail. Another potential avenue of research could involve comparative analyses across languages to determine if these meso-level properties are also observed in other languages.

### Conflict of interest statement

The author declares that the research was conducted in the absence of any commercial or financial relationships that could be construed as a potential conflict of interest.

## Footnotes

^1^The definition of *Q* may be appear to be similar to clustering coefficient, *C*, but they describe very different concepts. *C* measures the extent to which neighbors of a node are also neighbors of each other, and is a micro-level measure because it is computed at the level of individual nodes, whereas *Q* is a meso-level measure that is used to indicate the robustness of community structure of a network. See Borge-Holthoefer and Arenas (2010) for a discussion on the distinction between micro- and meso-levels of a network.

^2^The maximum value of 1.0 is achieved when the network consists of an infinite number of disjointed cliques (independent groups of nodes that do not connect to each other), and the mathematical proof can be found in Fortunato and Barthelemy (2007). Modularity values that are close to 1.0 generally indicate that community structure is a robust feature of the network.

^3^The Phonotactic Probability Calculator is available at www.people.ku.edu/~mvitevit/PhonoProbHome.html

^4^As suggested by a reviewer, an alternative baseline could be constructed by generating 100 sets of random communities and computing 95% confidence intervals of the lexical characteristics for each of the 17 communities to determine if the mean lexical characteristics of the real communities were significantly different from that of the random communities. The mean lexical characteristics for the 17 real communities did *not* fall within these confidence intervals [except for Community 11 (word frequency), Community 15 (neighborhood density) and Community 14 (age of acquisition)], which indicate that the pattern of results observed in the ANOVAs for the real network was not spurious and further support the results of the ANOVAs conducted on the 5 sets of random communities. A table of the confidence intervals of the 100 sets of random communities is included in Supplementary Materials.
