# Supplementary material for: Community structure in the phonological network
Source: Front Psychol. 2013 Aug 27;4:553. doi: 10.3389/fpsyg.2013.00553 (PMC3753538; doi:10.3389/fpsyg.2013.00553)
Supplement: Supplementary file 1 [file DataSheet1.PDF]

Supplementary Materials

(A) List of 10 most frequent biphones for the 17 real communities

Community																
1	2	3	4	5	6	7	8	9	10	11	12	13	14	15	16	17
ɨn	və	ɨs	ɪn	ɨt	ɹu	ʌn	ɪŋ	ɛn	ɑɹ	ɹi	st	nd	ɹɑɪ	ŋk	ɹe	oɹ
bɨ	ən	nɨ	nt	ən	ju	ʌm	læ	nt	bl̩	ɪk	tɚ	æn	ɑɪt	ɪŋ	en	ɹo
ɝb	ɪv	æɹ	ns	kɨ	lu	ɹʌ	lɪ	ɛl	mb	il	ɪs	ɹæ	æl	ɪl	tɹ	ol
ɪl	ɹɪ	ɹə	kt	æl	ut	gɹ	pl	ɛɹ	pɑ	ɑk	ɛs	kæ	ɑɪn	ɹɪ	et	st
ʤɨ	ət	ɹɨ	ɛk	lɨ	uz	pʌ	kl	ɹɛ	ɑp	in	ɹɪ	ɛn	li	wɪ	le	ko
ɝʤ	lɛ	əb	æn	mə	du	hʌ	fl	wɛ	wɑ	li	æs	æk	lɑɪ	ɪn	st	ɔl
tɝ	ɛv	bl̩	nf	ɹə	um	ʌb	lʌ	ɛd	ɑd	ik	ʌs	æm	sp	æŋ	ez	el
vɪ	ev	bə	ɝn	lə	tu	ʌt	sl	lɛ	ɑt	kɚ	lɪ	læ	tɹ	ɪɹ	ɛt	lo
ʒɨ	ɝs	əs	sɑɪ	ət	up	ni	ɪk	ɨn	bɚ	pi	lɚ	ns	ɑɪɹ	kw	ɪt	on
ɪb	ɛg	æb	ɛn	æɹ	un	ʌg	li	hɛ	ɑb	ni	ti	tæ	ɑɪd	ɪm	pɹ	to

(B) Table of confidence intervals of the 100 sets of random communities

Length

Community	Real Communities Mean length	Random Communities Lower bound	Upper bound
1	4.452	4.013	4.084
2	4.946	4.003	4.069
3	4.342	4.026	4.086
4	5.129	4.043	4.086
5	4.654	4.054	4.086
6	3.686	4.045	4.065
7	3.878	4.043	4.066
8	4.422	4.056	4.075
9	4.128	4.046	4.065
10	4.123	4.052	4.066
11	3.825	4.054	4.068
12	4.268	4.049	4.063
13	4.160	4.050	4.063
14	3.682	4.048	4.063
15	4.142	4.044	4.057
16	3.905	4.054	4.068
17	4.022	4.056	4.069

Subjective familiarity

Community	Real Communities Mean familiarity	Random Communities Lower bound	Upper bound
1	5.634	5.913	6.020
2	5.289	5.943	6.036
3	4.818	5.927	6.018
4	6.132	5.944	6.007
5	5.689	5.971	6.015
6	6.093	5.950	5.986
7	6.210	5.969	6.002
8	6.014	5.962	5.992
9	5.875	5.963	5.991
10	5.810	5.963	5.987
11	5.956	5.957	5.980
12	5.915	5.967	5.990
13	6.013	5.954	5.975
14	5.956	5.966	5.989
15	6.060	5.964	5.986
16	6.072	5.966	5.986
17	6.006	5.960	5.980

Word frequency

Community	Real Communities Mean frequency	Random Communities Lower bound	Upper bound
1	1.576	1.714	1.775
2	1.489	1.729	1.784
3	1.207	1.708	1.758
4	1.583	1.719	1.751
5	1.442	1.715	1.743
6	1.712	1.720	1.739
7	1.677	1.718	1.739
8	1.644	1.723	1.741
9	1.857	1.728	1.744
10	1.629	1.728	1.740
**11**	**1.729**	**1.727**	**1.740**
12	1.688	1.730	1.744
13	1.711	1.721	1.732
14	1.865	1.732	1.744
15	1.663	1.731	1.743
16	1.860	1.731	1.742
17	1.827	1.732	1.742

Neighborhood density

Community	Real Communities Mean density	Random Communities Lower bound	Upper bound
1	2.258	8.880	9.451
2	2.649	8.906	9.419
3	2.342	8.881	9.421
4	2.918	8.886	9.238
5	2.984	8.848	9.092
6	10.635	8.997	9.170
7	10.227	9.037	9.236
8	6.017	9.001	9.168
9	8.814	9.044	9.210
10	7.656	9.024	9.165
11	11.449	9.008	9.156
12	5.044	9.045	9.178
13	9.483	9.038	9.170
14	11.229	9.085	9.204
**15**	**9.096**	**9.021**	**9.139**
16	10.531	9.081	9.187
17	11.389	9.037	9.161

Neighborhood frequency

Community	Real Communities Mean NHF	Random Communities Lower bound	Upper bound
1	1.687	1.809	1.845
2	1.373	1.813	1.845
3	1.230	1.799	1.829
4	1.673	1.819	1.838
5	1.514	1.817	1.833
6	1.830	1.818	1.828
7	1.722	1.818	1.828
8	1.706	1.819	1.829
9	1.989	1.818	1.826
10	1.695	1.815	1.823
11	1.815	1.819	1.827
12	1.787	1.818	1.826
13	1.795	1.816	1.823
14	1.948	1.818	1.825
15	1.744	1.822	1.828
16	1.944	1.820	1.827
17	1.953	1.818	1.825

Segment probability

Community	Real Communities Mean segment probability	Random Communities Lower bound	Upper bound
1	0.04406	0.04674	0.04777
2	0.05677	0.04645	0.04745
3	0.04527	0.04624	0.04715
4	0.05092	0.04663	0.04726
5	0.06086	0.04681	0.04731
6	0.03885	0.04685	0.04721
7	0.04359	0.04686	0.04715
8	0.04662	0.04691	0.04718
9	0.05455	0.04690	0.04717
10	0.04627	0.04683	0.04705
11	0.04384	0.04698	0.04721
12	0.05089	0.04682	0.04706
13	0.05206	0.04691	0.04712
14	0.04164	0.04700	0.04722
15	0.04861	0.04690	0.04713
16	0.04282	0.04692	0.04713
17	0.04730	0.04701	0.04723

Biphone probability

Real Communities Random Communities

Community	Mean biphone probability	Lower bound	Upper bound
1	0.00471	0.00413	0.00433
2	0.00648	0.00398	0.00417
3	0.00354	0.00394	0.00411
4	0.00930	0.00402	0.00416
5	0.00638	0.00406	0.00416
6	0.00273	0.00406	0.00413
7	0.00309	0.00405	0.00411
8	0.00405	0.00409	0.00414
9	0.00545	0.00409	0.00414
10	0.00426	0.00408	0.00412
11	0.00288	0.00409	0.00414
12	0.00553	0.00407	0.00412
13	0.00526	0.00407	0.00411
14	0.00268	0.00410	0.00415
15	0.00449	0.00410	0.00414
16	0.00290	0.00409	0.00414
17	0.00396	0.00409	0.00413

Age of Acquisition	Real Communities	Random Communities	
Community	Mean age of acquisition	Lower bound	Upper bound
1	10.533	9.063	9.307
2	10.393	8.984	9.176
3	11.781	9.022	9.234
4	10.538	9.027	9.181
5	10.597	9.008	9.141
6	8.898	9.104	9.184
7	8.215	9.081	9.167
8	9.374	9.064	9.134
9	9.028	9.070	9.142
10	9.347	9.072	9.127
11	8.878	9.078	9.135
12	9.697	9.070	9.123
13	8.953	9.095	9.147
14	**9.091**	**9.067**	**9.115**
15	8.847	9.089	9.142
16	8.829	9.078	9.125
17	8.944	9.074	9.118

Note: The bolded lines indicate that the mean value lies within the confidence interval.

(C) Modularity values and number of communities at different resolution levels

	Resolution = 1.0		Resolution = 2.0		Resolution = 3.0		Resolution = 4.0		Resolution = 5.0	
Run	*N*	*Q*	*N*	*Q*	*N*	*Q*	*N*	*Q*	*N*	*Q*
1	29	0.668	**17**	**0.655**	10	0.614	6	0.412	5	0.025
2	25	0.670	15	0.678	9	0.556	7	0.397	7	0.072
3	30	0.673	19	0.674	10	0.554	5	0.507	9	0.025
4	27	0.679	19	0.665	12	0.631	5	0.437	6	0.021
5	26	0.686	18	0.662	11	0.589	6	0.318	6	0.042
Mean	27.4	0.675	17.6	0.667	10.4	0.589	5.8	0.414	6.6	0.037
